# Skin Barrier Modulation and Survival-Relevant Outcomes: A Systematic Review of Topical Emollients in Preterm Infants

**DOI:** 10.7759/cureus.105137

**Published:** 2026-03-12

**Authors:** Abdulelah F Alshehri, Mohammad Majrashi, Abdulmohsen Alanazi, Khalid Alshammari, Rana Alhazzani, Meshal Alzakari, Ayad A Bagazi, Abdullatif Aldhafyan, Faisal Alqarni, Khalid Almubarak, Bandar M Alotaibi, Osama Alhammad, Alwaleed Anab, Sultan AlMusfir, Alanoud Z Aljarbou

**Affiliations:** 1 College of Medicine, Imam Mohammad Ibn Saud Islamic University (IMSIU), Riyadh, SAU; 2 Department of Neonatology, Al Yamamah Hospital, Riyadh, SAU; 3 Department of Epidemiology, College of Health and Rehabilitation Sciences, Princess Nourah Bint Abdulrahman University, Riyadh, SAU; 4 Department of Pediatrics, College of Medicine, Imam Mohammad Ibn Saud Islamic University (IMSIU), Riyadh, SAU

**Keywords:** cutaneous colonization, neonatal skin integrity, skin barrier function, topical emollients, transepidermal water loss

## Abstract

Preterm neonates have an immature skin barrier that predisposes them to excessive water loss, microbial colonization, and systemic infection. Topical emollient therapy has been proposed as a low-cost strategy to support barrier function. However, the extent to which clinical benefits reflect direct permeability changes versus downstream biological effects remains unclear.

A systematic review was conducted in accordance with PRISMA 2020. PubMed, the Cochrane Library, and Google Scholar were searched from inception to January 31, 2025. Randomized or quasi-randomized controlled trials evaluating topical emollients in preterm neonates and reporting direct or proxy skin barrier-related outcomes were included. Due to substantial heterogeneity, results were synthesized narratively following the Synthesis Without Meta-analysis (SWiM) framework. Risk of bias was assessed using Risk of Bias Tool 2 (ROB-2), and certainty of evidence was evaluated using GRADE.

Seven randomized studies met the inclusion criteria. Only one preterm trial directly measured transepidermal water loss (TEWL) and demonstrated a significant reduction with coconut oil. Three trials reported improved neonatal skin condition scores (NSCS) with emollient therapy. Proxy outcomes included reduced skin deterioration, altered microbial profiles, and lower rates of culture-proven sepsis. A reduction in neonatal mortality was observed in one large trial conducted in a low-resource hospital setting. Heterogeneity in populations, emollient formulations, and outcome measures precluded quantitative meta-analysis. Overall certainty of evidence ranged from low to moderate.

Topical emollients are associated with improved skin condition and selected clinical outcomes in preterm neonates, despite limited direct evidence of barrier permeability modification. Observed clinical benefits appear context-dependent and are largely driven by a single large trial. Multidimensional trials integrating biophysical, microbiological, and clinical outcomes are needed to clarify mechanisms and guide targeted clinical application.

## Introduction and background

Preterm neonates enter extrauterine life with a developmentally constrained skin barrier that continues to mature throughout the early neonatal period. Globally, preterm birth affected an estimated 13.4 million live births in 2020, and its complications remain among the leading causes of neonatal and under-five mortality [1]. Despite major advances in neonatal intensive care, prematurity continues to impose a substantial global health burden, particularly in low- and middle-income countries, where infection-related morbidity is highest [2].

The epidermal barrier of preterm neonates is structurally immature, characterized by a thinner stratum corneum, reduced intercellular lipids, and impaired homeostatic regulation, resulting in elevated transepidermal water loss (TEWL) and increased permeability compared with term infants [3]. These alterations are most pronounced at lower gestational ages and contribute to excessive fluid and heat loss, delayed barrier maturation, and heightened susceptibility to environmental and microbial insults [4].

Beyond its mechanical role, neonatal skin functions as an active immunological interface, and barrier immaturity has been linked to dysregulated innate immune signaling and altered early-life microbial colonization, factors that increase the risk of cutaneous and systemic infection in preterm populations [5].

Topical emollient therapy has been proposed as a biologically plausible, low-cost strategy to support barrier integrity in preterm neonates. Emollients may enhance epidermal function by reducing TEWL, improving hydration, and supplementing deficient surface lipids, thereby stabilizing the stratum corneum and limiting microbial penetration [6,7].

Contemporary neonatal skin care research increasingly evaluates these effects using both direct biophysical measurements and bedside skin integrity scores [8]. However, the relative contribution of direct barrier improvement versus downstream clinical effects remains incompletely defined.

Evidence from randomized trials and contemporary meta-analyses suggests that routine topical emollient oil massage may be associated with a lower risk of invasive infection in preterm infants, particularly in low-resource settings and when applied repeatedly over prolonged periods [9]. In contrast, evidence from high-resource neonatal intensive care units has not consistently demonstrated reductions in serious infection or mortality outcomes [10].

These discrepancies highlight substantial contextual heterogeneity and suggest that the clinical impact of emollient therapy may depend on baseline infection risk, emollient formulation, and care environment. Additional clinical outcomes have also been explored. A recent systematic review and meta-analysis reported that topical emollient oil application was associated with improved postnatal weight gain in preterm newborns, indicating potential broader physiologic effects beyond the skin barrier alone [11]. Nevertheless, these associations are not uniformly observed across settings, and causal pathways linking barrier modulation to systemic outcomes remain uncertain.

Collectively, current evidence underscores the need to distinguish between direct measures of skin barrier function, proxy indicators of skin integrity, and downstream clinical endpoints. Heterogeneity in emollient formulations, trial designs, and outcome definitions continues to limit comparability across studies. A structured synthesis that integrates these outcome domains and contextualizes findings across diverse neonatal care settings is therefore required to inform evidence-based neonatal skin care practices.

## Review

Methods

Study Design and Reporting Framework

This study was conducted as a systematic review to evaluate the effects of topical emollient therapy on direct and proxy indicators of skin barrier function in preterm neonates. The review was reported in accordance with the PRISMA 2020 guidelines. Given the substantial clinical and methodological heterogeneity across the included studies and the small number of trials per outcome, quantitative meta-analysis was not appropriate. Therefore, findings were synthesized narratively following the principles of the Synthesis Without Meta-analysis (SWiM) framework, with results grouped by outcome domain. The review protocol was prospectively registered in the PROSPERO database (CRD420261298756). Ethical approval was not required, as this study was based exclusively on previously published data.

Eligibility Criteria

Eligible studies were randomized or quasi-randomized controlled trials that evaluated topical emollient therapy in preterm neonates (<37 weeks’ gestation) and reported at least one direct or proxy skin barrier-related outcome. Topical emollients were defined as any lipid-based preparation applied to the skin for preventive or therapeutic purposes. Comparator groups were required to receive standard neonatal skin care, massage-only care, or no emollient therapy.

Studies were excluded if they were observational, non-comparative, animal-based, in vitro, review articles, conference abstracts, or if they included only term neonates, evaluated systemic or non-topical interventions, or lacked extractable data. Physiological pilot or mechanistic randomized studies were retained only when they reported direct biophysical skin barrier outcomes.

Information Sources and Search Strategy

A comprehensive electronic search was conducted in PubMed, the Cochrane Library, and Google Scholar. The final search was performed on January 31, 2025, and covered records from database inception to the search date. The search strategy combined controlled vocabulary and free-text keywords related to prematurity, topical emollients, and skin barrier outcomes. Reference lists of included studies and relevant reviews were manually screened to identify additional eligible trials.

Study Selection

All retrieved records were imported into reference management software and duplicate records were removed. Two reviewers independently screened titles and abstracts to identify potentially relevant studies. Full-text articles of potentially eligible studies were retrieved and assessed for inclusion according to predefined criteria. Disagreements were resolved through discussion and consensus.

Data Extraction

All retrieved records were imported into reference management software and duplicate records were removed. Two reviewers independently screened titles and abstracts to identify potentially relevant studies. Full-text articles of potentially eligible studies were retrieved and assessed for inclusion according to predefined criteria. Disagreements were resolved through discussion and consensus.

Outcome Classification

Outcomes were categorized into direct and proxy indicators of skin barrier function to reflect both biophysical and clinically relevant dimensions of neonatal skin integrity. Direct outcomes comprised TEWL and validated neonatal skin condition scores (NSCS), which provide quantitative measures of epidermal barrier performance. Proxy outcomes included skin integrity trajectories, microbial colonization, culture-proven sepsis, and mortality, which were considered downstream or indirect indicators of compromised skin barrier function. This classification framework was used to structure the narrative synthesis and to interpret the findings across heterogeneous study designs and outcome measures.

Risk of Bias Assessment

Risk of bias was assessed using the Cochrane Risk of Bias Tool 2 (ROB-2). Each study was evaluated across five domains: randomization process, deviations from intended interventions, missing outcome data, measurement of outcomes, and selection of the reported result. Two reviewers independently conducted the assessments and disagreements were resolved by consensus.

Data Synthesis

Because of heterogeneity in participant characteristics, emollient formulations, intervention protocols, and outcome measurement tools, statistical pooling was not appropriate. Results were synthesized narratively and grouped according to outcome domain in line with SWiM guidance.

Certainty of Evidence

The certainty of evidence for each outcome domain was assessed using the GRADE approach, considering risk of bias, inconsistency, indirectness, imprecision, and potential publication bias.

Results

Screening of Included Studies

The systematic search identified a total of 236 records from PubMed, the Cochrane Library, and Google Scholar. No records were identified through registers. After the removal of 84 duplicate records, 152 records remained for title and abstract screening. Of these, 127 records were excluded for not meeting the eligibility criteria.

A total of 25 full-text articles were sought for retrieval, and all were successfully retrieved. These 25 reports were assessed for eligibility. Of these, 18 reports were excluded for the following reasons: ineligible population (n = 7), non-comparative study design (n = 4), absence of relevant skin barrier outcomes (n = 5), and insufficient data reporting (n = 2).

**Figure 1:**
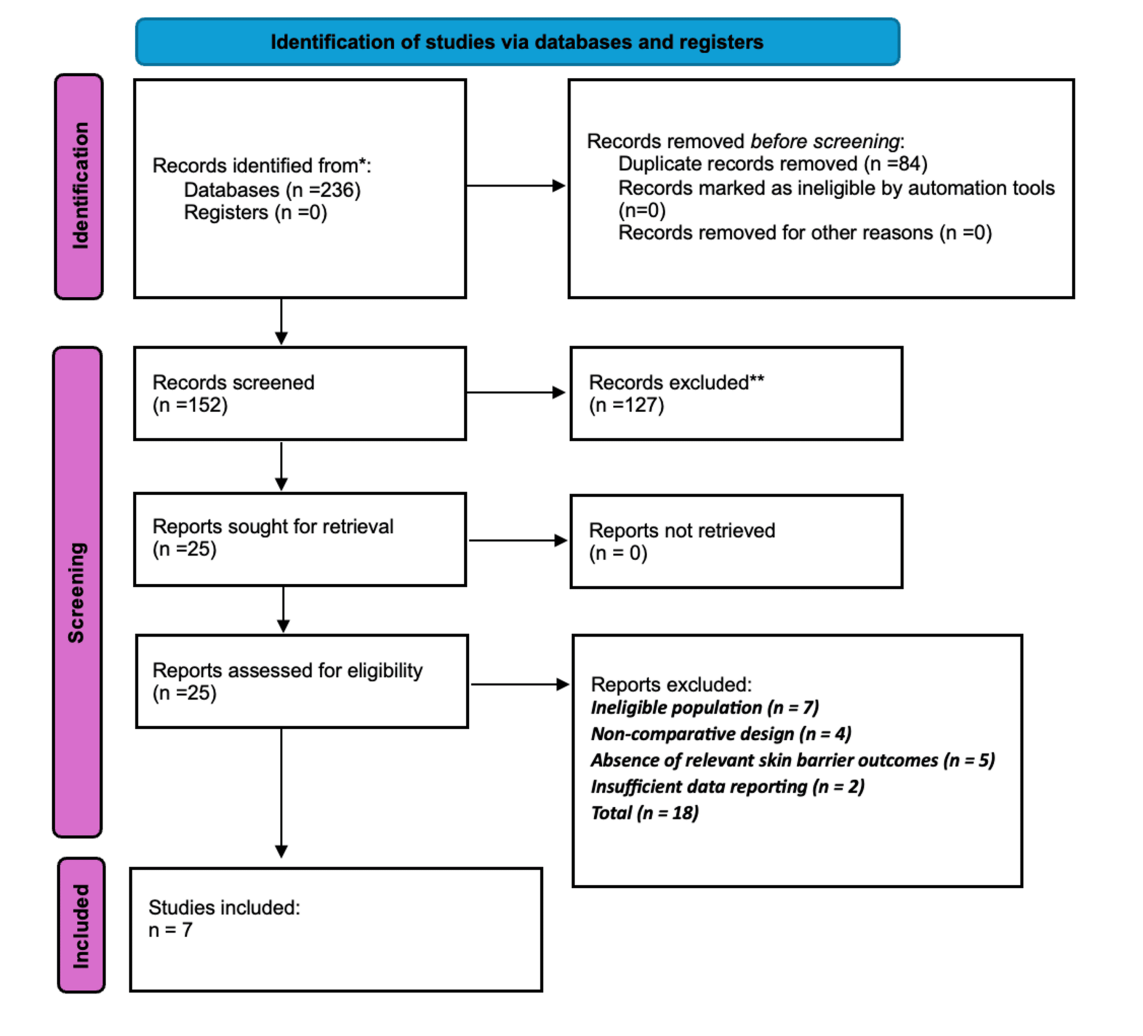
PRISMA flow diagram of study selection

Consequently, seven studies were included in the final systematic review. The study selection process is summarized in the PRISMA flow diagram (Figure 1).

Study Characteristics

Seven randomized controlled studies were included in this review. The trials were conducted across diverse geographic settings, predominantly in low- and middle-income countries, including India and Bangladesh, with one study conducted in a high-income neonatal intensive care unit in Australia and one physiological pilot study in the United Kingdom. The study populations ranged from very low birth weight and very preterm infants (<30 weeks’ gestation) to moderate preterm infants (<37 weeks’ gestation), with one study conducted exclusively in full-term neonates. Two trials directly quantified skin barrier function using biophysical measures of TEWL or validated skin condition scores (NSCS). One additional randomized trial assessed skin condition using a validated NSCS as the primary outcome. The remaining studies evaluated indirect or proxy indicators of skin barrier integrity, including skin condition trajectories, microbial composition, bloodstream infection, and mortality. Interventions consisted primarily of topical natural oils, most commonly coconut oil or sunflower seed oil, applied to the whole body, with or without massage. One study compared olive oil and sunflower oil with no oil in healthy term neonates. Most trials initiated emollient therapy within the first 12-24 hours of life, although formulations, frequency, and duration varied substantially. Control groups received standard neonatal skin care or no emollient therapy (Table 1).

**Table 1:** Characteristics of included studies (n = 7) TEWL, Transepidermal Water Loss; NSCS, Neonatal Skin Condition Score; VLBW, Very Low Birth Weight; LOS, Length of Stay

Study	Country	Setting	Design	Gestational Age	Emollient	Comparator	Barrier Measurement Type	Primary Outcomes	Timing of Initiation
Nangia et al. (2015) [12]	India	NICU	RCT	VLBW, median 32 wks	Coconut oil	Standard care	Direct	TEWL, skin score, colonization	12 h of life
Strunk et al. (2018) [13]	Australia	NICU	RCT	<30 wks	Coconut oil	Standard care	Direct	NSCS, skin irritation	<24 h
Ghori et al. (2023) [14]	Australia	NICU	RCT (sub-study)	<30 wks	Coconut oil	Standard care	Indirect	Skin microbiome, LOS episodes	<24 h
Konar et al. (2019) [15]	India	Community + hospital	RCT	<37 wks	Coconut oil	Massage only	Indirect	NSCS, sepsis, hypothermia, neurodevelopment	Early neonatal period
Darmstadt et al. (2008) [16]	Bangladesh	Hospital	RCT	<33 wks	Sunflower oil / Aquaphor	No emollient	Indirect	Mortality, sepsis	≤72 h of life
Darmstadt et al. (2014) [17]	Bangladesh	Hospital	Secondary RCT analysis	<33 wks	Sunflower oil / Aquaphor	No emollient	Indirect	Skin score trajectory, infection risk	≤72 h of life
Cooke et al. (2016) [18]	UK	Maternity ward	RCT (pilot)	Full-term	Olive oil / Sunflower oil	No oil	Direct (physiologic)	Lipid lamellae, TEWL, hydration	Within 72 h

Transepidermal Water Loss (TEWL)

Only one randomized controlled trial directly quantified TEWL as a primary outcome in preterm neonates using a validated biophysical device. In the study by Nangia et al. [12], TEWL was measured every 12 hours during the first week of life using a closed-chamber evaporimeter (Table 2). Very low birth weight preterm infants who received twice-daily topical coconut oil demonstrated a statistically significant reduction in TEWL compared with standard care (mean difference 6.80 g/m²/h, 95% CI 3.48-10.15; p < 0.01).

**Table 2:** Direct biophysical measurements of TEWL TEWL, Transepidermal Water Loss; VLBW, Very Low Birth Weight; ATR-FTIR, Attenuated Total Reflectance - Fourier Transform Infrared Spectroscopy

Study (ref)	Population	Device	Timepoints	Outcome Type	Main Finding
Nangia et al. (2015) [12]	VLBW preterm (median GA 32 weeks)	Closed-chamber evaporimeter	Every 12 h until day 7	Direct TEWL	Statistically significant reduction in TEWL in the coconut oil group
Cooke et al. (2016) [18]	Full-term neonates	Biox Aquaflux Model AF200 + ATR-FTIR	Baseline and 4 weeks	Direct TEWL	Trend toward improved skin barrier function (pilot study)

In the OBSeRvE pilot trial by Cooke et al. [18], TEWL was measured in healthy full-term neonates at baseline and at four weeks, using a closed-chamber device (Biox Aquaflux Model AF200) alongside Attenuated total reflectance - Fourier transform infrared spectroscopy (ATR-FTIR) (Table 2). No significant differences in TEWL were observed between the intervention and control groups, and the study was not powered for clinical efficacy outcomes.

No other included study directly measured TEWL using validated biophysical instruments. Therefore, evidence for direct barrier permeability outcomes is limited to a single interventional trial in preterm neonates, and quantitative synthesis was not feasible. Narrative synthesis was undertaken in accordance with SWiM guidance.

Skin Integrity and Condition Scores

Three included trials assessed neonatal skin integrity using either validated skin condition scores or structured skin trajectory analyses. In the randomized controlled trial by Strunk et al. [13], very preterm infants (<30 weeks’ gestation) receiving topical coconut oil demonstrated significantly better NSCS over 21 days, compared with standard care (p = 0.01). Similarly, Nangia et al. [12] reported improved skin condition scores during the first week of life among very low birth weight preterm infants treated with coconut oil. In a secondary analysis of a large randomized controlled trial, Darmstadt et al. [17] examined skin condition trajectories and demonstrated that deterioration in skin scores was significantly lower in both emollient groups (sunflower seed oil and Aquaphor), compared with controls (p < 0.05). Variation in scoring systems, follow-up intervals, and reporting formats precluded quantitative pooling. However, all three studies consistently reported improved or preserved skin condition among emollient-treated neonates compared with controls (Table 3).

**Table 3:** Skin integrity and condition outcomes following topical emollient application

Study	Assessment Tool	Emollient	Comparator	Outcome Type	Main Finding
Strunk et al. (2018) [13]	Neonatal Skin Condition Score (NSCS)	Coconut oil	Standard care	Direct skin score	Significantly better NSCS in the emollient group (p = 0.01)
Nangia et al. (2015) [12]	Validated skin condition score	Coconut oil	Standard care	Direct skin score	Improved skin condition during the first week
Darmstadt et al. (2014) [17]	Skin condition score trajectory	Sunflower seed oil / Aquaphor	No emollient	Indirect proxy	Lower rate of skin deterioration in emollient groups (p < 0.05)

Cutaneous Colonization

Three included trials reported microbiological or infection-related skin outcomes using culture-based methods or structured safety monitoring. In the randomized controlled trial by Nangia et al. [12], skin swab cultures were obtained, and a significantly higher proportion of infants in the coconut oil group demonstrated sterile cultures at day 7, compared with standard care. In the large randomized controlled trial by Darmstadt et al. [16], emollient-treated infants (sunflower seed oil or Aquaphor) experienced significantly lower rates of culture-proven bloodstream infection. This trial did not directly quantify skin surface colonization but reported systemic infection outcomes. In Strunk et al. [13], skin infection and irritation were monitored as safety outcomes, and no increase in infectious complications was observed in the coconut oil group compared with controls. Across studies, topical emollient therapy was not associated with increased cutaneous infection. However, evidence for a direct effect on skin microbial colonization remains limited and heterogeneous (Table 4).

**Table 4:** Microbiological and infection-related skin outcomes following topical emollient therapy

Study	Method	Emollient	Comparator	Main Outcome
Nangia et al. (2015) [12]	Skin swab cultures	Coconut oil	Standard care	Higher proportion of sterile cultures at day 7
Darmstadt et al. (2008) [16]	Blood cultures (sepsis)	Sunflower seed oil / Aquaphor	No emollient	Reduced rates of culture-proven bloodstream infection
Strunk et al. (2018) [13]	Safety monitoring (skin infection)	Coconut oil	Standard care	No increase in infectious complications

Secondary Outcomes

Sepsis: Two included trials reported culture-confirmed bloodstream infection as a clinical outcome. In the large randomized controlled trial by Darmstadt et al. [17], preterm neonates receiving topical sunflower seed oil or Aquaphor experienced significantly lower rates of culture-proven bloodstream infection, compared with controls. In the randomized trial by Konar et al. [15], late-onset sepsis was reported as a secondary outcome, with a lower incidence observed in the coconut oil group, compared with the massage-only control group. In Strunk et al. [13], sepsis was monitored as a secondary safety outcome, and no significant differences were observed between groups. Although these findings suggest an association between topical emollient therapy and reduced infection risk, only the Darmstadt trial was adequately powered to evaluate clinical infection endpoints.

Mortality: Mortality was reported in one large randomized controlled trial. In the study by Darmstadt et al. [17], treatment with sunflower seed oil resulted in a 26% reduction in neonatal mortality, while Aquaphor reduced mortality by 32%, compared with standard care. No other included study was powered to assess mortality outcomes.

Adverse skin events: Adverse skin events were infrequently reported across studies. In Strunk et al. [13] and Nangia et al. [12], mild, transient skin irritation was observed in a small number of infants, with no serious adverse reactions attributed to emollient use. No study reported treatment discontinuation or clinically significant dermatitis related to topical therapy. Overall, topical emollient therapy was well tolerated in the included trials.

Subgroup Patterns (Qualitative)

Qualitative cross-study comparison identified several consistent patterns. These observations were hypothesis-generating only and were not derived from formal statistical subgroup analyses. Earlier initiation of emollient therapy (within the first 24-48 hours of life) was associated with more consistent improvements in skin condition and infection-related outcomes, compared with later initiation. The type of emollient appeared to influence outcomes. Natural vegetable oils, particularly coconut oil and sunflower seed oil, demonstrated more consistent benefits across both barrier-related and clinical endpoints than synthetic formulations. The degree of prematurity also appeared to modify treatment response. Very and extremely preterm infants (<32 weeks’ gestation) demonstrated larger relative improvements than more mature preterm infants, although this observation was inferred rather than directly tested. These qualitative patterns should be interpreted with caution because of heterogeneity in populations, interventions, and outcome measures.

Risk of Bias (RoB-2)

**Figure 2:**
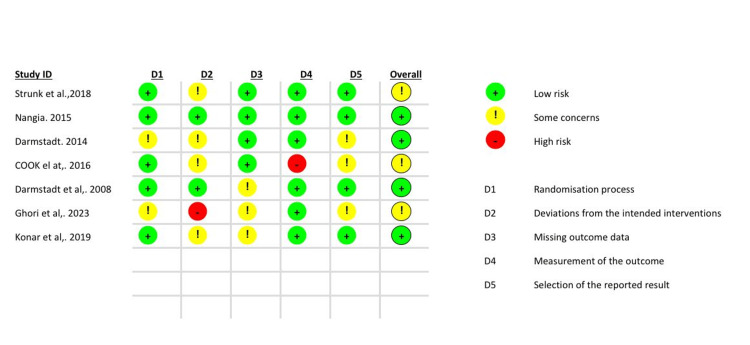
Risk of bias summary (RoB-2) assessment across seven randomized clinical trials Studies included: Strunk et al. (2018) [13], Nangia et al. (2015) [12], Darmstadt et al. (2014) [17], Cooke et al. (2016) [18], Darmstadt et al. (2008) [16], Ghori et al. (2023) [14], Konar et al. (2019) [15]

Overall, the included studies demonstrated low- to moderate risk of bias across most domains. Low risk across most RoB-2 domains was observed in Nangia et al. [12], Strunk et al. [13], and Darmstadt et al. [16], reflecting adequate randomization and outcome assessment. Some concerns were identified in domains related to deviations from intended interventions (D2) and selection of the reported result (D5), particularly in trials with open-label designs or secondary analyses. Cooke et al. [18] and Ghori et al. [14] demonstrated a higher risk of bias in at least one domain, because of pilot design, lack of blinding, or selective outcome reporting, which may limit internal validity. Overall, the risk of bias across the included studies was judged as low to moderate (Figure 2).

Certainty of Evidence (GRADE)

The certainty of evidence for each outcome was assessed using the GRADE framework. Risk of bias judgments were informed by ROB-2. Certainty ratings considered risk of bias, inconsistency, indirectness, imprecision, and publication bias. Given the small number of studies per outcome, publication bias could not be formally assessed. Overall, the certainty of evidence ranged from low to moderate, primarily due to imprecision, heterogeneity in outcome measures, small sample sizes, and reliance on indirect clinical proxies (Table 5).

**Table 5:** GRADE summary of findings for topical emollient therapy in preterm neonates TEWL, Transepidermal Water Loss; NSCS, Neonatal Skin Condition Score

Outcome	Number of Studies	Study Design	Downgrade Rationale (GRADE Domains)	Certainty
TEWL	1	RCT	Imprecision (single small trial); indirectness (one study only)	Low
Skin integrity / NSCS	3	RCTs	Risk of bias; indirectness (proxy scoring in one trial)	Low-Moderate
Cutaneous colonization	1	RCT	Indirectness (single culture-based study); imprecision	Low
Sepsis	3	RCTs	Indirectness (secondary outcome in 2 trials); imprecision	Low
Mortality	1	RCT	Imprecision (single trial); indirectness (not a dermatologic endpoint)	Low

Discussion

This synthesis identifies a clinically relevant signal for topical emollient therapy in preterm neonates; however, this signal is derived predominantly from proxy and clinical endpoints rather than from direct biophysical measures of barrier permeability. Only one included preterm trial directly quantified TEWL, and, therefore, direct evidence for permeability modification remains limited. The discordance between sparse TEWL data and reported improvements in infection- and survival-related outcomes indicates that conventional water-flux metrics alone may not fully represent neonatal skin barrier function [19]. This observation should be interpreted descriptively rather than causally.

Contemporary dermatologic frameworks describe neonatal skin as a multifunctional interface extending beyond permeability to include lipid architecture, antimicrobial surface properties, and immune-modulatory functions [3,20]. Within this context, TEWL reflects only one component of barrier performance and may not capture pathways relevant to microbial translocation or inflammatory susceptibility. Accordingly, the absence of consistent TEWL improvement across trials does not exclude biologically meaningful effects, but highlights the limitations of relying on a single surrogate marker [19].

Clinical outcome patterns must be interpreted with caution. Reductions in bloodstream infection and mortality are largely driven by a single large, randomized trial conducted in a low-resource hospital setting. This dominance limits external validity and precludes inference of a universal treatment effect. In contrast, trials conducted in high-resource neonatal intensive care units did not demonstrate corresponding reductions in sepsis or mortality, despite reporting improvements in skin condition scores. These findings indicate heterogeneity in treatment response that cannot be resolved by the current evidence base.

Environmental and contextual factors may modify observed effects; however, such interpretations remain speculative within this dataset. Differences in baseline infection risk, hygiene infrastructure, and environmental microbial burden may influence clinical outcomes, consistent with broader evidence on the global epidemiology of neonatal infections [21]. Nevertheless, no trial-level stratification was available to test this hypothesis directly.

Evidence from experimental and clinical microbiome research demonstrates that neonatal skin microbial communities evolve dynamically and may associate with infection risk, particularly in preterm populations [22]. While these data provide biological plausibility, the reviewed trials did not concurrently measure biophysical, microbiological, and clinical endpoints within the same cohorts. Consequently, no mediational pathway linking topical emollient use to systemic outcomes can be established from the available evidence.

Physiological studies further caution against generalization. Neonatal barrier maturation is highly dependent on lipid organization and structural development, processes that may be differentially affected by exogenous lipid exposure depending on gestational maturity [20]. Moreover, immunologic-barrier interactions in neonatal skin differ from those of older infants and adults, reinforcing the need to interpret emollient effects within a developmental context [3].

This review integrates direct, proxy, and clinical outcome domains to provide a multidimensional appraisal of neonatal skin barrier-related effects. The structured narrative synthesis, aligned with SWiM principles, enhances transparency and minimizes overreliance on any single surrogate outcome. The inclusion of studies across diverse care settings further allows contextual interpretation of heterogeneity.

Several limitations constrain inference. Most trials relied on indirect or subjective outcome measures, and direct TEWL data were available from only one preterm study. Substantial heterogeneity in emollient formulations, application protocols, and outcome definitions precluded quantitative synthesis. Additionally, a single large trial disproportionately influences infection and mortality outcomes, limiting generalizability. Finally, the absence of concurrent measurement of permeability, microbiology, and clinical endpoints within individual studies restricts mechanistic interpretation.

## Conclusions

This review reframes topical emollient therapy from a supportive skin care practice to a potential biological modifier of neonatal vulnerability. Across seven randomized trials, emollients were consistently associated with improved skin condition and selected clinical outcomes, even in the absence of robust biophysical confirmation of barrier permeability change. The concentration of survival benefits within a single large, low-resource trial underscores both the promise and the contextual dependence of this intervention. Collectively, these findings shift the focus from whether emollients work to how, and in whom, they may be most effective. Future research must move beyond single-parameter outcomes and adopt integrated designs that simultaneously assess skin biology, microbial dynamics, and clinical endpoints, to define mechanism-informed, setting-specific neonatal skin care strategies.
